# A Confined Replacement Synthesis of Bismuth Nanodots in MOF Derived Carbon Arrays as Binder‐Free Anodes for Sodium‐Ion Batteries

**DOI:** 10.1002/advs.201900162

**Published:** 2019-06-17

**Authors:** Yifang Zhang, Qiong Su, Wenjie Xu, Guozhong Cao, Yaping Wang, Anqiang Pan, Shuquan Liang

**Affiliations:** ^1^ Department of Materials Physics and Chemistry School of Materials Science and Engineering Central South University Changsha Hunan 410083 China; ^2^ Department of Chemistry and Energy Sciences Institute Yale University West Haven CT 06516 USA; ^3^ Department of Materials Science and Engineering University of Washington Seattle WA 98195 USA; ^4^ Department of Materials Physics and Chemistry Light Alloy Research Institute Central South University Changsha Hunan 410083 China

**Keywords:** binder‐free anodes, bismuth, carbon, metal–organic frameworks

## Abstract

The inferior tolerance with reversible accommodation of large‐sized Na^+^ ion in electrode materials has plagued the adaptability of sodium‐ion chemistry. The sluggish diffusion kinetics of Na^+^ also baffles the desirability. Herein, a carbon fiber supported binder‐free electrode consisting of bismuth and carbon composite is designed. Well‐confined bismuth nanodots are synthesized by replacing cobalt in the metal–organic frameworks (MOF)–derived, nitrogen‐doped carbon arrays, which are demonstrated with remarkable reversibility during sodiation and desodiation. Cobalt species in the pristine MOF catalyze the graphitization around organic components in calcination, generating a highly conductive network in which the bismuth is to be embedded. The uniformly dispersed bismuth nanodots provide plenty boundaries and abundant active sites in the carbon arrays, where fast sodium storage kinetics are realized to contribute extra capacity and excellent rate performance.

## 1 Introduction

As a potential substitute for currently commercialized lithium‐ion batteries, the sodium‐ion battery is a promising battery technology for low‐cost and large‐scale electric energy storage because of the abundant sodium reserves.^1^ The larger Na^+^ ion than Li^+^ ion (1.02 Å vs 0.76 Å), however, makes it awkward to accommodate Na^+^ in suitable host electrode materials, especially anode ones.^2^ More efforts for remedying the limitation of anode materials are still required.

Among the studied anode materials for Na^+^ batteries, hard carbon is plagued by low capacity and inferior Coulombic efficiency.^3^ Metal chalcogenides^4^ with conversion reaction and some layered compounds^5^ with insertion reaction often suffer from low electronic conductivity. Moreover, they are usually working at relatively high potential and experiencing large discharge/charge voltage hysteresis that would compromise the energy density and efficiency.^6^ Metals and alloys have high electronic conductivity, while the alloying/dealloying with sodium ineluctably leads to continuous pulverization that may detach the active material from current collector and deteriorate the cyclability.^7^, ^8^ To address these issues, reducing the particle to nanosize is desired for easy release of the strain generated during sodiation/desodiation.^9^ However, bare nanoparticles would undergo aggregation. A conductive skeleton, usually carbon matrix, that holds the active particles can restrain aggregation and further buffer the volume change. Nevertheless, high temperature is often needed in synthesizing such composite to carbonize the precursor that loads the active materials. During this process, the active particles may experience heat agglomeration.^10^ Therefore, it is still challenging to uniformly disperse ultrafine nanoparticles in a carbon scaffold.

Metal–organic frameworks (MOFs), as an emerging class of crystalline porous materials, are constructed by well‐organized metal centers and organic linkers.^11^ The pyrolytic organic species can serve as carbon skeletons after annealing in inert atmosphere.^8^, ^12^ The organic ligands of some MOFs may contain some desired elements, which can be used to produce heteroatom‐doped carbon materials.^13^ With the catalysis of some metal species (Co, Ni, and Fe), the pyrolytic organic parts can be further graphitized.^14^, ^15^, ^16^ Thanks to the strong and reticular linking of inorganic and organic units in MOF crystals, researchers have utilized various MOFs as template to uniformly disperse nanosized particles in a matrix.^17^

Here we report a replacement synthesis route to confine ultrafine bismuth nanodots in MOF derived carbon host. Cobalt organic framework arrays are deposited on carbon fibers in an aqueous solution, which are calcinated to cobalt metal confined in carbon arrays afterward. The carbon fibers afford high surface to distribute the arrays that avoid the collapse of the templates and largely reduces the heat agglomeration of cobalt. Bismuth nanodots are then confined in the carbon arrays by replacing cobalt, which offer good tolerance with the accommodation of Na^+^. In addition, the boundaries between bismuth nanodots and carbon provide abundant active sites, that contribute extra capacity with fast Na^+^ diffusion kinetics. The as‐prepared bismuth nanodots and carbon composites demonstrate low working potential, highly reversible capacity, and good rate capability as binder‐free anode materials for sodium‐ion batteries.

## 2 Results and Discussion

**Figure 1:**
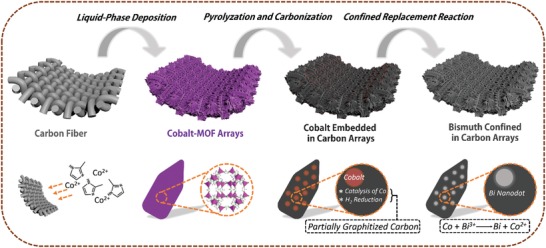
Schematic illustration of the synthesis of bismuth nanodots confined in carbon arrays.

The fabrication process of bismuth nanodots confined in carbon arrays is illustrated in **Figure** **1**. Carbon fibers are used as flexible substrates to support the active materials. Cobalt organic framework arrays are first grown on carbon fibers via a facile liquid‐phase deposition approach at room temperature.^18^ After the deposition, cobalt MOFs in purple color uniformly cover the surface of carbon fiber, with good adhesion even when the carbon fiber is under high bending state (Figure S1, Supporting Information). After annealing in 95% Ar and 5% H_2_ atmosphere at 800 °C, the Co^2+^ species are reduced to metallic Co, embedded in carbon arrays that are derived from the organic species. The carbon arrays still attach firmly on the carbon fiber after calcination, as the carbon substrate can be attracted easily by a magnetic stirrer owing to the Co on it (Figure S1, Supporting Information). Under the reduction of H_2_ atmosphere and the catalysis of Co, the carbon arrays are with high graphitization, which is beneficial for fast electron transportation (Figure 1). After the confined replacement reaction, bismuth nanodots in situ occupy the position of Co nanodots. The bismuth nanodots are well dispersed in the carbon arrays, which can shorten the pathways for ionic diffusion and mitigate the strain during sodiation/desodiation.

**Figure 2:**
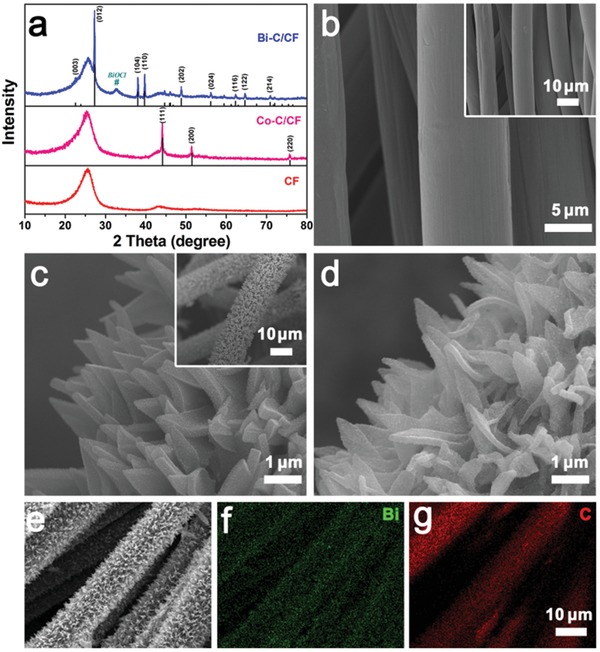
a) XRD patterns of bare CF, Co–C/CF, and Bi–C/CF. SEM images of b) bare CF, c) Co–C/CF, and d) Bi–C/CF. e–g) Elemental mapping images of Bi–C/CF.

**Figure** **2**a displays the X‐ray powder diffraction (XRD) pattern of blank carbon fiber, which shows a bulged peak at around 26°. The fibers are around 8 µm in diameter with smooth surface (Figure 2b). After depositing Co‐MOF on the fibers, the XRD pattern shows additional peaks in accordance with pure Co‐MOF (Figure S2a, Supporting Information), implying the successful growth of Co‐MOF on the carbon fiber substrate.^19^ From the scanning electron microscopy (SEM) images (Figure S2b, Supporting Information), it can be seen that the Co‐MOF arrays uniformly cover the carbon fibers. The total diameter of one fiber in Co‐MOF/CF is around 15–18 µm, implying the height of arrays is around 3.5–5 µm. An enlarged SEM image (Figure S2c, Supporting Information) shows the arrays are around 200–400 nm in thickness with smooth surface. In contrast, bare Co‐MOF flakes synthesized without the support of carbon fiber are nonuniform and incomplete, with sizes ranging from 1 to more than 10 µm (Figure S2d, Supporting Information). After calcination, the arrays still stick firmly on the carbon fiber (inset of Figure 2c), while their surface is much rough comparing to that of the MOF precursor. Some particles can be observed dispersing uniformly on the surface (Figure 2c), which originate from the cobalt species in the MOFs being reduced to cobalt metal (XRD pattern, Figure 2a). The cobalt particles on the surface may be relatively larger owing to aggregation caused by the high surface reaction activity. However, the aggregation is restrained to the minimum level benefiting from the well‐distributed carbon arrays, that guarantee least contacts between the cobalt particles in the carbon matrix. Without the carbon fiber substrate, in contrast, the cobalt particles may grow very large, as shown in the SEM image of the annealed product from bare Co‐MOF (Figure S3, Supporting Information).

Although some cobalt particles on the surface of the arrays may dissolve in the bismuth chloride solution, the cobalt inside the arrays can displace bismuth ions, leaving bismuth substance in its original site. The XRD pattern of the product after the replacement reaction shows diffraction peaks of metallic bismuth (Figure 2a). A small impurity peak can be identified as BiOCl, which is resulted from the hydrolysis of trace unwashed BiCl_3_. As shown in Figure 2d, the arrays on the carbon fibers can maintain their shapes after the replacement reaction. Energy‐dispersive X‐ray spectroscopy (EDS) result implies cobalt is completely replaced by bismuth (Figure S4, Supporting Information). Moreover, the elemental mapping results (Figure 2e–g) reveal the homogeneous distribution of bismuth on the carbon fibers, further verifying the successful synthesis of bismuth confined in carbon arrays under the support of carbon fiber substrate. The weight ratio of Bi in the composite arrays is estimated to be 49.3% by thermogravimetric analysis (TG) analysis, which is shown and discussed in Figure S5 in the Supporting Information. In comparison, the product after replacement reaction from bare Co–C composite is not with good uniformity or structural integrity. The original shape of flakes can barely be recognized (Figure S6, Supporting Information). This may be caused by the heterogeneous sizes and nonuniform distributions of cobalt in the arrays, which may easily destroy the structure during the replacement reaction. In addition, there are some small particles between the tattered pieces, which are BiOCl originating from unwashed BiCl_3_, as verified by XRD and elemental mapping result (Figures S6 and S7, Supporting Information). The BiCl_3_ cannot be removed as easily as in the carbon fiber supported sample, where the impurities can be rinsed even in a vigorous intensity without flushing out the arrays. Bare metallic bismuth is also prepared using cobalt powders as the replacement reaction reagent. As shown in Figure S8 in the Supporting Information, the products are irregular particles with sizes ranging from 0.8 to 1.5 µm, and BiOCl impurities also exist in the sample. These results imply the advantages of making bismuth and carbon composite supported by carbon fibers to obtain pure and well‐designed products.

**Figure 3:**
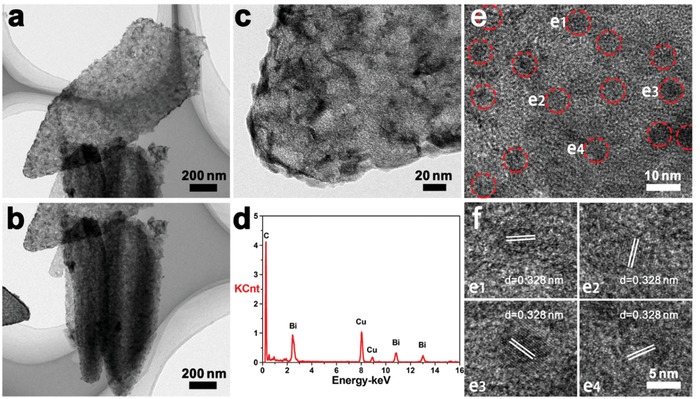
a–c) TEM images, d) EDS result, and e,f) HRTEM images of Bi–C/CF.

Transmission electron microscopy (TEM) images provide detailed observations of the arrays detached from the carbon fiber by high power ultrasonic. **Figure** **3**a shows a flake with length of around 1.5 µm and width of around 0.7 µm. The two flakes in Figure 3b are standing against each other and the thickness of one flake can be observed to be 200–400 nm. There are many pits around the surface of the flake, resulting from the removal of cobalt nanoparticles that were attached (Figure 3c). The different contrast on the edge of the pits may be owing to the original cobalt particles formed during high temperature reduction, which subsequently catalyze the graphitization of around carbons.^15^, ^20^ As verified by high‐resolution transmission electron microscopy (HRTEM) image in Figure S9 in the Supporting Information, lattice fringe with *d*‐spacing of 0.354 nm can correspond to planar distance of (002) plane of graphitized carbon. The interlayer distance of the partially graphitized carbon matrix is larger than graphite (0.335 nm),^21^ which can be ascribed to the defects and nitrogendoping in the carbon arrays. The EDS result implies the existence of bismuth and no cobalt signal can be detected (Figure 3d). Copper signals are from the TEM grid. In the HRTEM image of Figure 3e, some nanodots with size of about 5 nm can be found dispersing well in the carbon matrix. The enlarged pictures of some areas in Figure 3e are given in Figure 3f, which show lattice fringes with *d*‐spacings of 0.328 nm, corresponding to the planar distance of (012) plane of bismuth. These results demonstrate cobalt would not experience aggregation inside the carbon arrays and its replacement product, bismuth, can also be well confined in the carbon matrix with nanoscale size. The confined replacement strategy is thus considered very reliable to avoid the aggregation of active materials.

**Figure 4:**
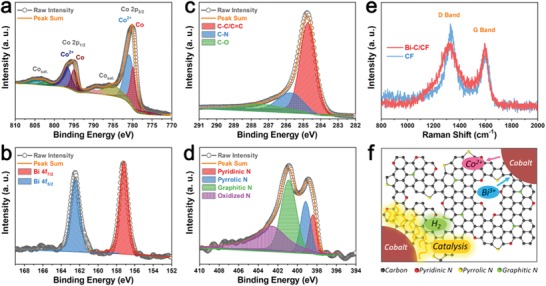
a) Co 2p high‐resolution XPS spectra of Co–C/CF. b) Bi 4f, c) C 1s, and d) N 1s high‐resolution XPS spectra of Bi–C/CF. e) Raman spectra of Bi–C/CF and blank carbon fiber. f) Schematic structure of partially graphitized, nitrogen‐doped carbon derived from the Co‐MOF, as well as the synthesis of bismuth and carbon composite by confined replacement reaction.

XPS was conducted to further investigate the chemical composition of Co–C/CF and Bi–C/CF (Figure S10, Supporting Information). Co, C, N, and O elements are detected in the survey spectra of Co–C/CF. The cobalt nanoparticles on the surface of arrays lead to strong cobalt peaks. O element is detected which may be owing to the partial oxidation of nanosized cobalt and the adsorbed oxygen as the sample was exposed to air. While in the spectra of Bi–C/CF, the relative intensity of O peak is much reduced comparing to that of C peak, and no cobalt signal is detected. Owing to the confinement of carbon, the peaks of Bi are also not with high intensity because XPS is only sensitive to several nanometers beneath the surface. Co 2p high resolution XPS spectra of Co–C/CF are shown in **Figure** **4**a. The peaks at 780.8 and 796.6 eV correspond to Co 2p_3/2_ and Co 2p_1/2_ for Co^2+^, respectively, with ∆*E* = 15.8 eV close to CoO (∆*E* = 15.5 ± 0.1 eV). The peaks at 779.9 and 795.0 eV are Co 2p_3/2_ and Co 2p_1/2_ peaks for metallic Co, with ∆*E* = 15.1 eV close to Co (∆*E* = 15.05 eV).^22^ The other deconvolution peaks can be assigned to the satellite peaks of Co 2p. The XPS result implies that, despite the inevitable surface oxidation which generates some CoO, cobalt species in Co‐MOF are reduced to metallic Co after calcination in inert atmosphere. The Bi 4f spectra of Bi–C/CF show standard peaks that agree with reported ones (Figure 4b).^23^ In Figure 4c, the C 1s spectra indicate that carbon is doped with heteroatoms, which can be ascribed to nitrogen derived from the organic species in Co‐MOF, as depicted in the high resolution N 1s spectra of Bi–C/CF (Figure 4d). The composition of the carbon matrix originates from the Co–C/CF sample, as analyzed by the C 1s and N 1s XPS tests, with Co–C/CF showing the same nitrogen doping features (Figure S11, Supporting Information). The carbon matrix is with relatively high graphitization, verified by the sharp G Raman band at around 1590 cm^−1^ that shows its graphitic feature comparing with carbon fiber or other kinds of carbon (Figure 4e).^24^ The broad D band at about 1330 cm^−1^ implies the existence of abundant defects, which may come from nitrogen doping, as well as the boundaries with bismuth nanodots. As illustrated in Figure 4f, the partial graphitization of the carbon matrix can be ascribed to the catalysis effect of cobalt under the reduction of H_2_ atmosphere.^14^, ^15^ Different kinds of nitrogen (pyridinic, pyrrolic, or graphitic) doping on carbon induces a large number of defects. Moreover, the replacement of Co by Bi would not change the composition and structure. The boundaries between carbon and bismuth generate more defects and active sites. These features endow the carbon matrix with fast electron and Na^+^ ion transfer kinetics, good adsorbent ability for Na^+^ ions, and improved surface capacitive effects.

**Figure 5:**
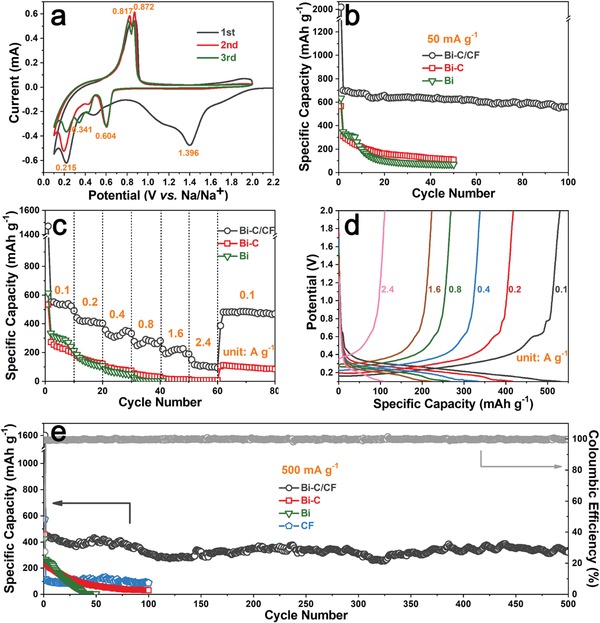
a) The initial three successive CV curves of Bi–C/CF at scan rate of 0.1 mV s^−1^ between 0.1 and 2 V. b) Cycling performance of Bi–C/CF, Bi–C, and bare Bi at 50 mA g^−1^. c) Rate performance of Bi–C/CF, Bi–C, and bare Bi. d) Discharge/charge profiles of Bi–C/CF at different current densities. e) Long‐term cycling performance of Bi–C/CF, Bi–C, bare Bi, and bare carbon fiber at 500 mA g^−1^.

The Bi–C/CF samples were evaluated as binder‐free electrodes for sodium‐ion batteries. Cyclic voltammetry (CV) was first carried out to investigate the reaction between Na and Bi–C/CF. The selected voltage range is between 0.1 and 2 V to minimize the impact of carbon fiber, which is illustrated in Figures S12–14 in the Supporting Information and discussed in the supporting information. In the initial scan of Bi–C/CF electrode from open circuit to 0.1 V (**Figure** **5**a), the broad cathodic peak at 1.396 V is related to the formation of solid electrolyte interphase (SEI) on the carbon arrays and carbon fiber substrate. The other cathodic peak at 0.215 V can be ascribed to the activation of bismuth to form sodium alloys and formation of SEI on Bi.^25^ The anodic peaks at 0.817 and 0.872 V correspond to the multistep dealloying process of Na*_x_*Bi to Bi. In the following cycles, no peak for SEI on carbon is observed, while the peaks related to activation of bismuth are still found because some Bi nanodots are confined deeply in the carbon arrays. Besides, a cathodic peak at 0.341 V gradually appears along with the shrinkage of the activation peak. Other than that, the rest peaks are well overlapped. The two sets of peaks located at 0.604/0.872 and 0.341/0.817 V correspond to the reversible formation of NaBi and Na_3_Bi, which are quite consistent with previous reports.^26^

Figure 5b reveals the cycling performances of Bi–C/CF, Bi–C, and bare Bi electrodes at 50 mA g^−1^. The initial three discharge/charge processes for the Bi–C/CF electrode are given in Figure S15 in the Supporting Information, with all platforms corresponding well to the CV peaks. The Bi–C/CF electrode can maintain a high capacity of around 630 mAh g^−1^ during cycling. The capacity is higher than theoretical capacity of pure bismuth (385 mAh g^−1^),^27^ which can be ascribed to some capacity contribution of carbon arrays as well as the carbon fiber substrate. The Bi–C sample only delivered 308.7 mAh g^−1^ of capacity in the second cycle and only 108.9 mAh g^−1^ was reserved after 50 cycles. Without the support of carbon fibers that uniformly distribute the carbon arrays, the Bi–C sample is with less active surface. The bismuth particles are not well confined and more likely to pulverize, detach from the conductive skeleton, and lose activity after repeated sodiation/desodiation. While for the bare Bi electrode, although its initial capacities are higher than those of Bi–C, it shows even worse stability, implying the inferior structure of unprotected bismuth anode in terms of enduring the mechanical stress along cycling. The SEM image of the Bi–C/CF electrode after 100 cycles is shown in Figure S16 in the Supporting Information. Despite the shape changes owing to the SEI formed on the surface, the arrays are still well dispersed with firm connection to the carbon fiber. Figure 5c shows the rate performance of three samples. The Bi–C/CF electrode can deliver specific capacities of 550, 423, 338, 283, 227, and 110 mAh g^−1^ at current densities of 0.1, 0.2, 0.4, 0.8, 1.6, and 2.4 A g^−1^, respectively. Specific capacity of 481 mAh g^−1^ can be recovered when the current is reset to 0.1 A g^−1^. The discharge/charge profiles of Bi–C/CF at different rates demonstrated in Figure 5d are in good accordance with the CV curves. In Figure 5e, comparison of long‐term cycling of the samples at 500 mA g^−1^ further reveals the highest capacity and considerable stability with high Coulombic efficiency for the Bi–C/CF electrode. A bare carbon fiber cycled at the same current with Bi–C/CF was also given in Figure 5e, implying the carbon fiber substrate only contributes little capacity to the Bi–C/CF electrode. The performance of the Bi–C/CF electrode is compared with previously reported bismuth or its hybrid composite electrodes, which are summarized in **Table** **1**. This unique Bi–C/CF binder‐free electrode demonstrates superior properties as promising anode materials for sodium‐ion batteries.

**Table 1:** Electrochemical performances of bismuth‐based electrodes for sodium‐ion batteries

Electrode Description	Voltage Window [V]	Current Density [mA g^−^^1^]	Specific Capacity [mAh g^−^^1^]	Coulombic Efficiency [%]
Bismuth nanorod bundle^28^	0.1–2.0	50 500	350 211	Not mentioned
Bismuth^25^	0.1–2.1	50 800	394 371	98.7
Bi_0.57_Sb_0.43_‐C^29^	0.05–2.0	100 500	393 357	99.5
Bi@graphene^30^	0.01–2.0 0.3–0.9	40 40	561 358	Not mentioned
Bi/C nanofibers^31^	0.01–3.0	100 800	302 194	Near 100
Bi/carbon nanofibers^32^	0.01–2.0	50 500	379 112	98
Bi nanosheets on carbon fiber^33^	0.01–2.0	50 200	350 240	Near 100
Bi@C microsphere^34^	0.01–2.0	100 500	260 178	98
Bi nanospheres in porous carbon^35^	0.01–2.5	200	106	Near 100
This Work	0.1–2.0	50 100 800	630 550 283	99.7

**Figure 6:**
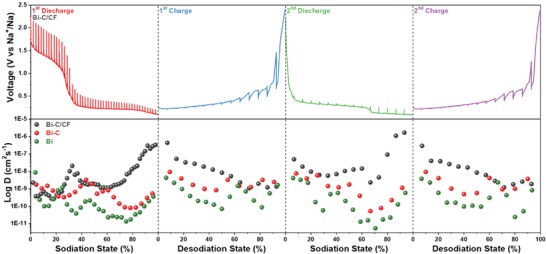
GITT curves of Bi–C/CF and corresponding Na^+^ diffusion coefficient at different sodiation states. The Na^+^ diffusion coefficients of Bi–C and Bi are also given for comparison.

To further inquire into the sodium storage kinetics of the Bi–C/CF electrode, galvanostatic intermittent titration technique (GITT) was employed to evaluate the Na^+^ diffusion and conductivity properties in different stages during the sodiation/desodiation process.^10^, ^36^ The detail of testing setup is illustrated in Figure S18 in the Supporting Information. The obtained GITT curves of Bi–C/CF for the first two discharge/charge processes are shown in **Figure** **6**, with the sodium diffusion coefficients at different sodiation/desodiation states calculated and plotted underneath. Comparing with Bi–C (Figure 6, Figure S19, Supporting Information), the Bi–C/CF electrode experienced lower Na^+^ diffusion coefficient at 0–20% sodiation states or 80–100% desodiation states. This could be owing to the well‐confined bismuth nanodots in the carbon arrays, which are less easy to be sodiated comparing with the aggregated and exposed bismuth particles in the Bi–C sample. However, the sodium diffusion coefficients increased much faster at 20–30% sodiation states for the Bi–C/CF, implying the higher diffusion rate in nanosized bismuth active materials. Moreover, the Bi–C/CF electrode showed high sodium diffusion at 70–100% sodiation states or 0–30% desodiation states, which can be ascribed to the capacitive sodium storage with high kinetics.^37^ As compared with the Bi–C sample, the well‐dispersed bismuth nanodots in the Bi–C/CF may create more defects in the carbon arrays, which are beneficial for the adsorption of Na^+^ ions that contributes some capacitive capacity to the overall sodium storage. The capacitive contribution to the overall capacity is further evaluated by CV measurements at different scan rates. As shown in Figure S20 in the Supporting Information, a high capacitive contribution ratio of 87.46% can be obtained at scan rate of 1 mV s^−1^, implying the capacitive Na‐storage occupies a great quantity of the whole capacity. This can be ascribed to the much‐enlarged surface area by uniformly distributing Bi–C arrays on carbon fiber substrate (Figure S21, Supporting Information).

## 3 Conclusion

In summary, a binder‐free electrode containing bismuth and carbon composite was successfully synthesized. A facile liquid‐phase deposition of MOF arrays on carbon fibers provides uniform and sturdy templates for subsequent calcination and replacement reactions. The carbon fibers afford high surface to distribute the arrays and avoid their collapse in further synthesis processes. The obtained bismuth nanodots confined in carbon arrays could well accommodate the volume changes during sodiation/desodiation. Additional capacity with fast Na^+^ diffusion kinetics is observed, thanks to the nitrogen‐doped carbon arrays that are abundant with active sites at the boundaries with bismuth nanodots.

## 4 Experimental Section

*Synthesis of Cobalt Organic Framework Arrays Grown on Carbon Fibers*: Cobalt nitrate hexahydrate (0.582 g) and 2‐methyimidazole (1.3 g) were separately dissolved in 40 mL of deionized water and stirred for 30 min. 1 × 1 cm^2^ carbon fiber (CF) pieces were then immerged into the Co(NO_3_)_2_ solution and sonicated until all air bubbles adsorbed on the CF escaped. Afterward, the 2‐methyimidazole solution was added. After stirring for uniform mixing, the obtained purple solution was kept still for 2 h. The CF pieces coated by cobalt metal–organic frameworks (Co‐MOF/CF) were then taken out, rinsed by deionized water, and finally dried for further usage. Cobalt metal–organic frameworks (Co‐MOF) powders were also synthesized without adding CF as supporting substrate. The Co‐MOF precipitates from the mixture solution were collected and washed with water by centrifugation.

*Synthesis of Bismuth Nanodots Confined in Carbon Arrays*: Co‐MOF/CF or Co‐MOF was annealed in a tube furnace at 800 °C for 2h with a heating rate of 2 °C min^−1^ under a gas flow composed of 95% Ar and 5% H_2_. The obtained products were cobalt nanoparticles confined in the carbon arrays, which were denoted as Co–C/CF or Co–C for the ones derived from Co‐MOF/CF or Co‐MOF, respectively. The as prepared Co–C/CF or Co–C was immerged into a mixture containing 0.5 mmol of bismuth chloride that dispersed in 35 mL of ethylene glycol/methanol (6:1 in volume) solvent. The above mixture was stirred overnight to allow the complete displacement reaction of cobalt with bismuth. Afterward, the carbon fibers coated by bismuth confined in carbon arrays (Bi–C/CF) were sonicated and rinsed with methanol. While the powders without the CF substrate were washed with methanol by centrifugation. The products were dried to obtain the final samples. Moreover, bare Bi powders were obtained by adding stoichiometric cobalt powder in bismuth chloride solution and stirred overnight, followed by the same washing and drying procedures.

*Materials Characterization*: The XRD patterns were recorded on a Rigaku D/max 2500 XRD (Cu Kα radiation, λ = 1.54178Å). The morphologies and structures of the samples were characterized by SEM(Nova NanoSEM230) and TEM(Titan G2 60‐300). TG analysis was conducted on NETZSCH STA 449C instrument. X‐ray photoelectron spectroscopy (XPS) was performed on ESCALAB 250Xi (ThermoFisher‐VG Scientific, Britain). Raman spectra were measured at room temperature on LabRAM Hr800. Nitrogen adsorption–desorption measurements were tested at 77 K on NOVA 4200e (Quantachrome Instruments).

*Electrochemical Measurement*: The Bi–C/CF pieces were used as binder‐free anodes, which were assembled into 2025‐type coin cells in a glovebox (Mbraun, Garching, Germany) filled with ultrahigh purity argon. The loading mass of Bi–C on the CF substrate was about 1–1.2 mg cm^−2^. Bi–C or bare Bi active materials were also made to working electrodes by mixing them with super‐p and carboxymethyl cellulose (CMC) in mass ratio of 8:1:1 in deionized water to make slurry, coating the slurry on copper foils and drying in vacuum. 1 mNaClO_4_ in propylene carbonate (PC) with 5% fluoroethylene carbonate (FEC) was used as the electrolyte. Na metal foil was used as the counter and reference electrode. Glass microfiber (Whatman, GF/D) was used as the separator. Cyclic voltammetry (CV) was tested with an electrochemical workstation (CHI660C). The galvanostatic charge/discharge performances of the electrodes were conducted at room temperature on a Land battery tester (Land CT 2001A, Wuhan, China). The sodium diffusion and conductivity properties were evaluated by GITT.

## Conflict of Interest

The authors declare no conflict of interest.

## Supporting information

Supplementary
