# Supplementary material for: A Confined Replacement Synthesis of Bismuth Nanodots in MOF Derived Carbon Arrays as Binder‐Free Anodes for Sodium‐Ion Batteries
Source: Adv Sci (Weinh). 2019 Jun 17;6(16):1900162. doi: 10.1002/advs.201900162 (PMC6702623; doi:10.1002/advs.201900162)
Supplement: Supplementary file 1 — Supplementary [file ADVS-6-1900162-s001.pdf]

Copyright WILEY-VCH Verlag GmbH & Co. KGaA, 69469 Weinheim, Germany, 2019.

Supporting Information

for *Adv. Sci.,* DOI: 10.1002/advs.201900162

A Confined Replacement Synthesis of Bismuth Nanodots in MOF Derived Carbon Arrays as Binder-Free Anodes for Sodium-Ion Batteries

Yifang Zhang, Qiong Su, Wenjie Xu, Guozhong Cao, Yaping Wang, Anqiang Pan,* and Shuquan Liang**

Copyright WILEY-VCH Verlag GmbH & Co. KGaA, 69469 Weinheim, Germany, 2019.

Supporting Information

A Confined Replacement Synthesis of Bismuth Nanodots in MOF Derived Carbon Arrays as Binder-Free Anodes for Sodium-Ion Batteries

Yifang Zhang, Qiong Su, Wenjie Xu, Guozhong Cao, Yaping Wang, Anqiang Pan* and Shuquan Liang**

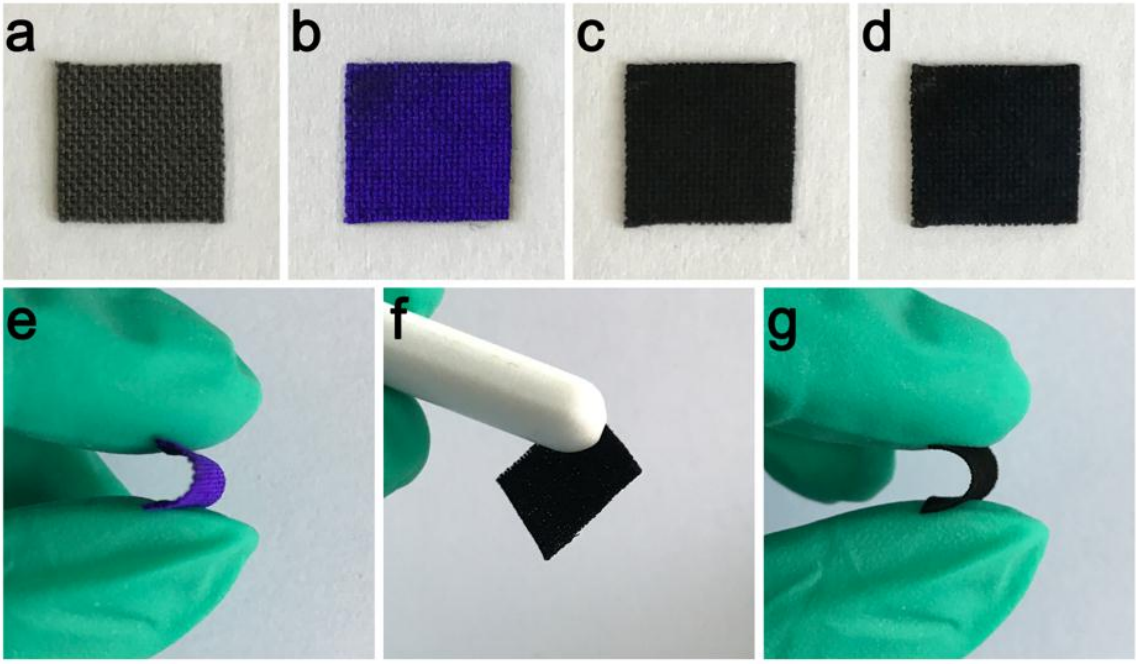

Figure S1. Photos of bare carbon fiber (a), Co-MOF/CF (b), Co-C/CF (c), and Bi-C/CF (d). A photo of Co-MOF/CF under bending (e). A photo of Co-C/CF attracted by a magnetic stirrer (f). A photo of Bi-C/CF under bending (g).

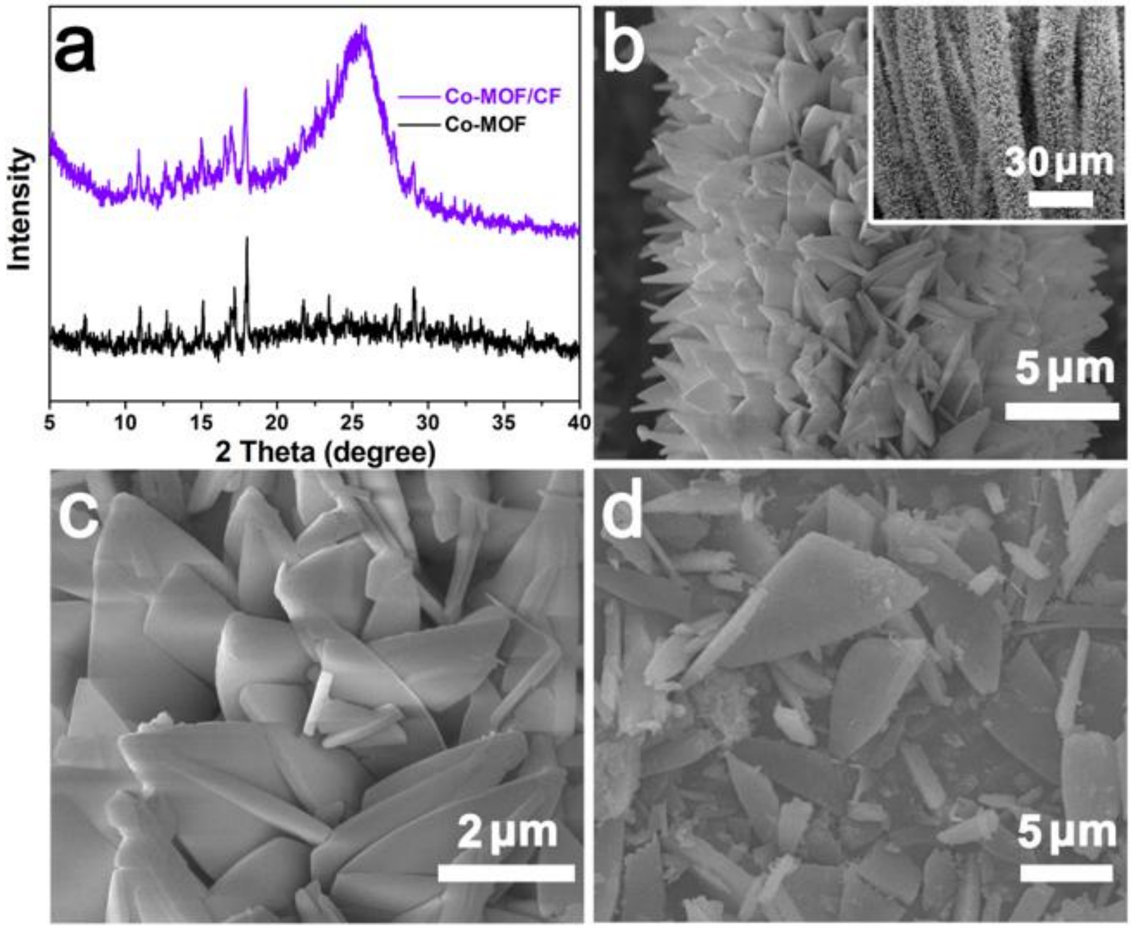

Figure S2. XRD patterns of Co-MOF/CF and Co-MOF (a). SEM images of Co-MOF/CF (b, c) and Co-MOF (d).

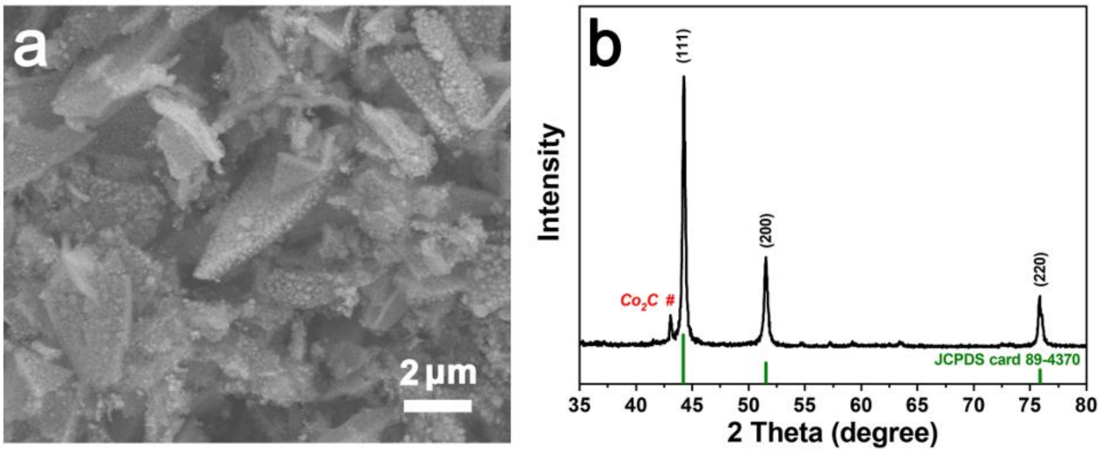

Figure S3. SEM image (a) and XRD patterns (b) of Co-C.

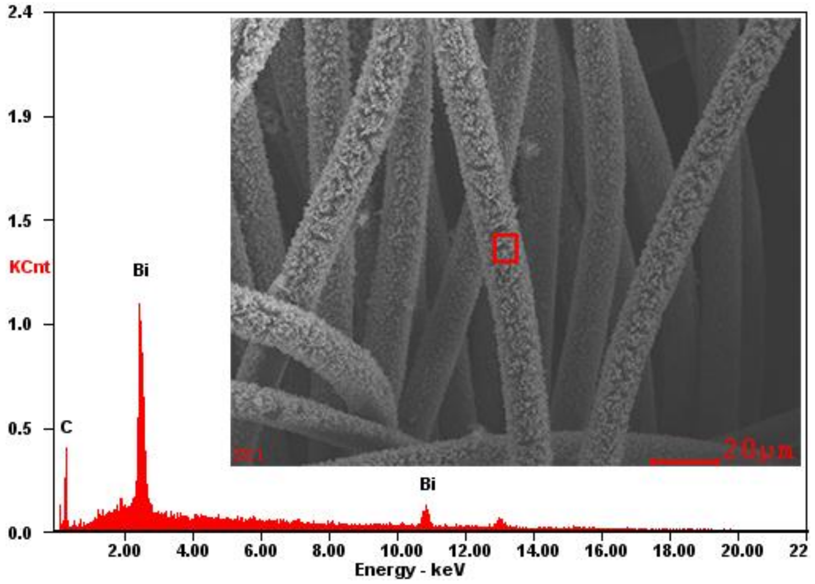

Figure S4. EDS result of Bi-C/CF.

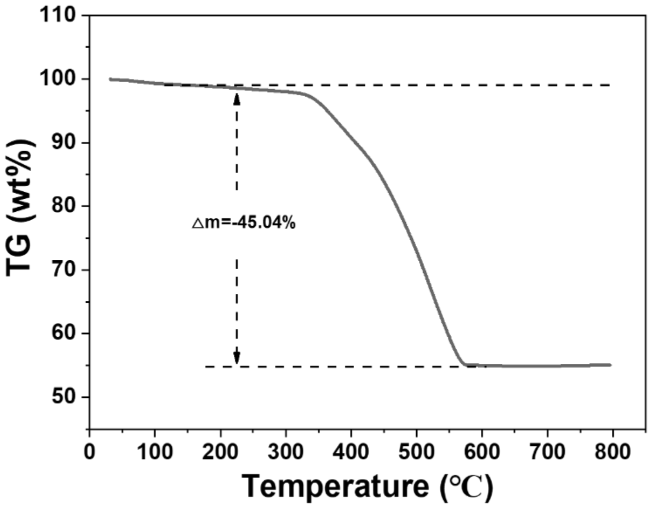

Figure S5. TG curve of Bi-C composite arrays obtained from Bi-C/CF.

To estimate the weight ratio of Bi in the composite arrays, the Bi-C composite arrays were detached from the carbon fiber by high power ultrasonic and collected for TG test under air atmosphere. As shown in Figure S5, during the heating process to 800 ℃, bismuth and carbon were oxidized to Bi_2O_3 and CO_2 gas, respectively.[1] The remaining 54.96% of pristine weight is from Bi_2O_3, implying the weight ratio of Bi is 49.3% in the composite arrays.

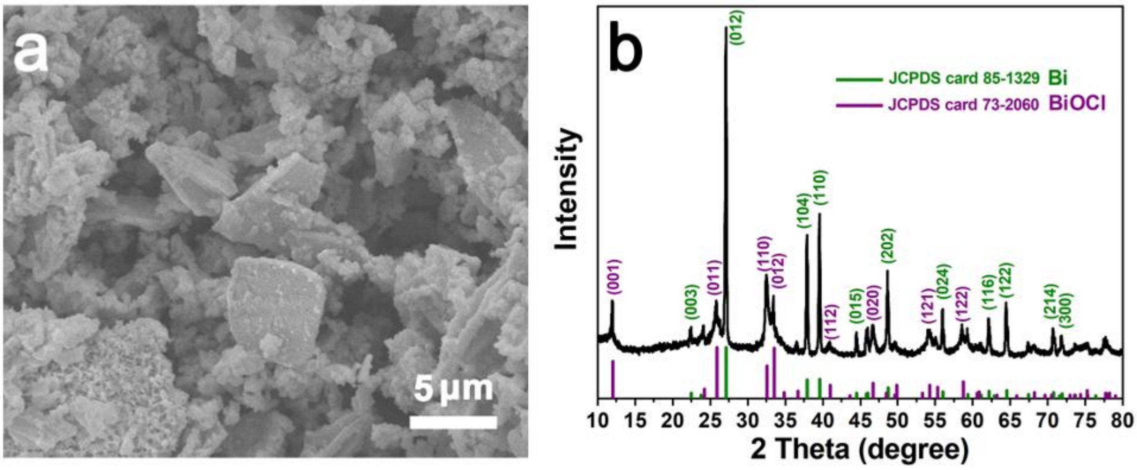

Figure S6. SEM image (a) and XRD patterns (b) of Bi-C.

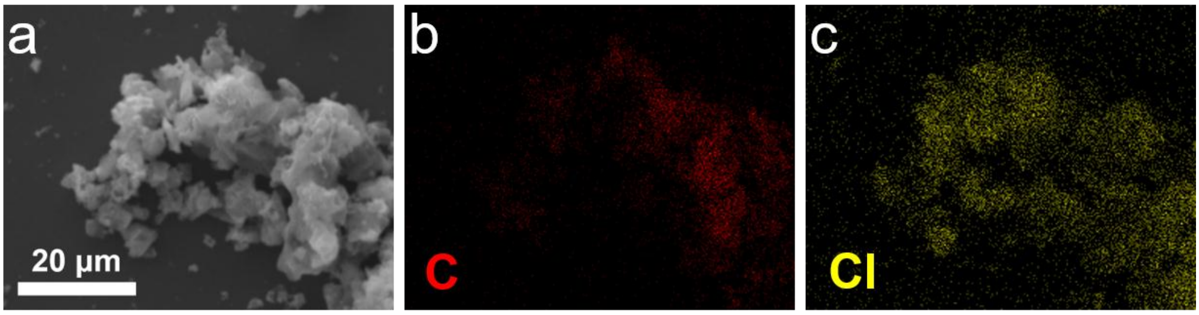

Figure S7. Elemental mapping images of Bi-C sample. The distributions of C and Cl elements are not uniform, implying the tattered Bi-C flakes are covered by some BiOCl particles.

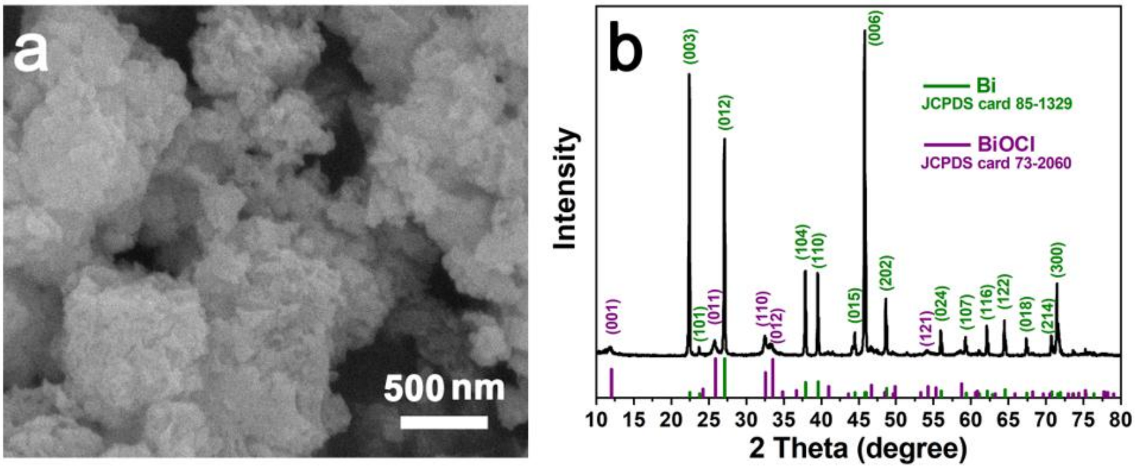

Figure S8. SEM image (a) and XRD patterns (b) of bare Bi.

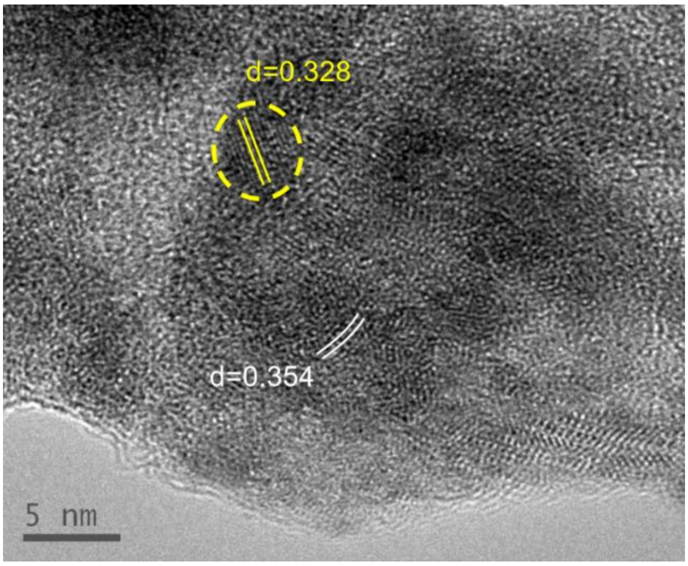

Figure S9. HRTEM image of Bi-C/CF. The lattice fringes with *d*-spacings of 0.328 and 0.354 nm correspond to planar distance of (012) plane of bismuth and (002) plane of graphitized carbon, respectively.

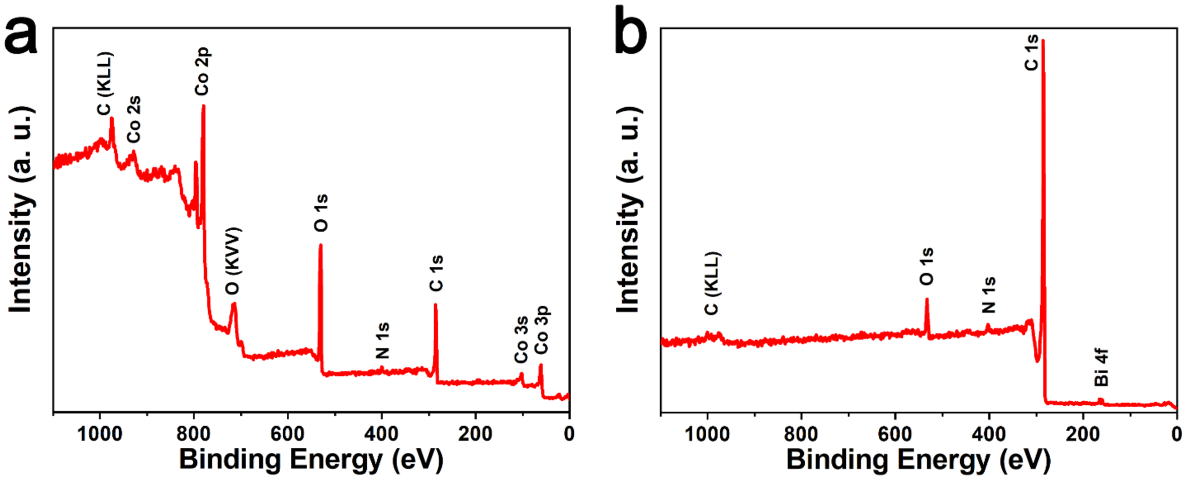

Figure S10. XPS survey spectra of Co-C/CF (a) and Bi-C/CF (b).

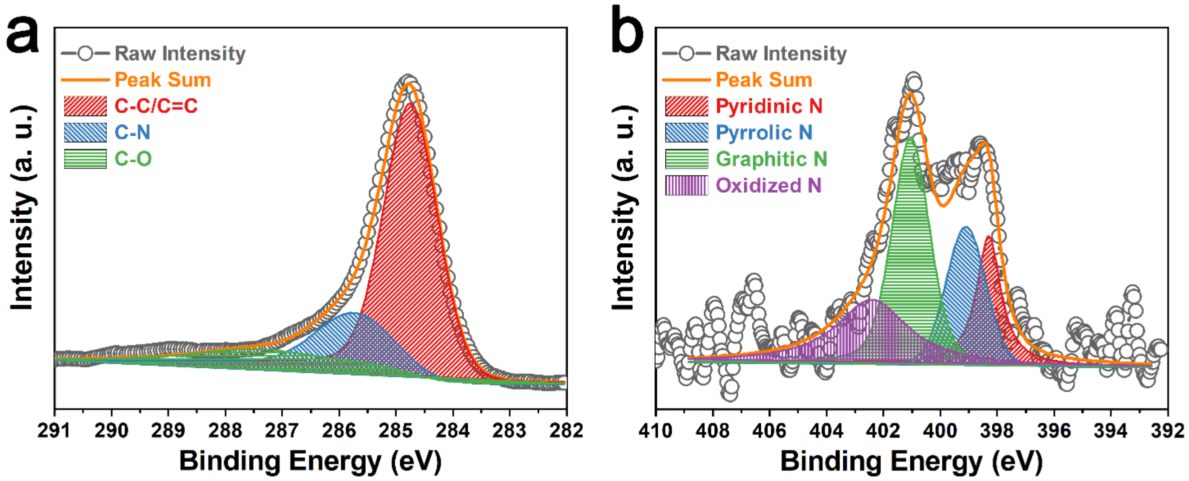

Figure S11. C 1s (a) and N 1s (b) high-resolution XPS spectra of Co-C/CF.

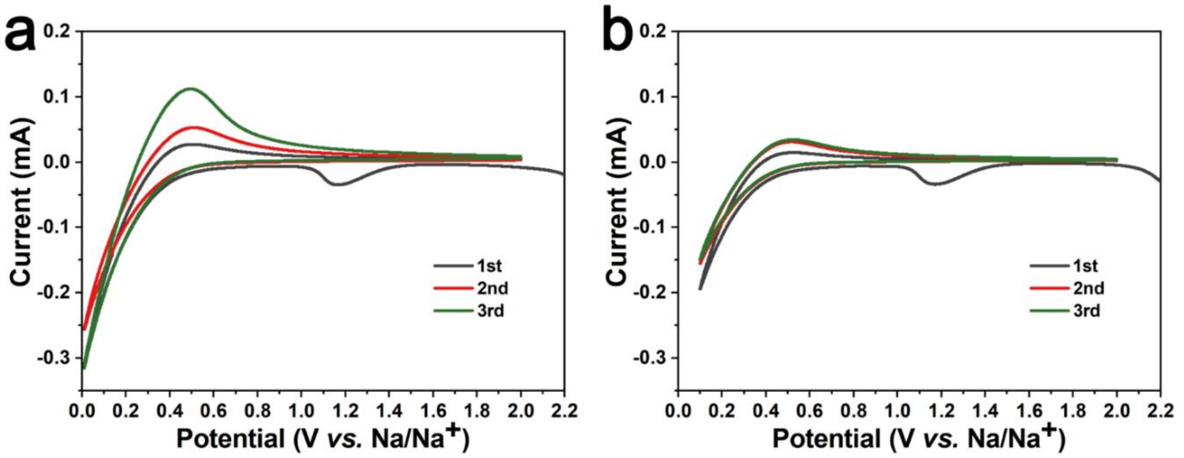

Figure S12. The initial three successive CV curves of bare carbon fiber at scan rate of 0.1 mV s^{-1} between 0.01-2 V (a) or 0.1-2 V (b).

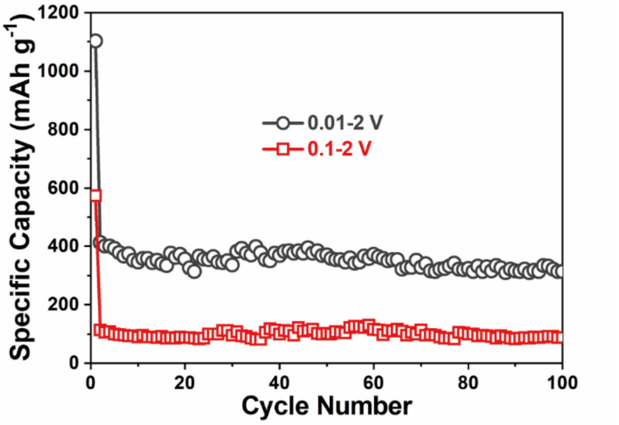

Figure S13. Cycling performance of bare carbon fiber between 0.01-2 V or 0.1-2 V. For convenient comparison with the as-synthesized Bi-C/CF samples, the specific capacities for bare carbon fibers are calculated assuming there are 1 mg of Bi-C arrays. The applied current is 0.5 A g^{-1}. In this way, the impact and contribution of carbon fibers to the capacity of Bi-C/CF can be easily evaluated.

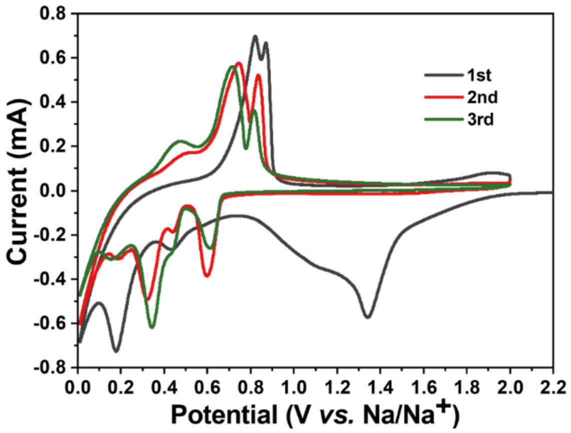

Figure S14. The initial three successive CV curves of Bi-C/CF at scan rate of 0.1 mV s^{-1} between 0.01-2 V.

As shown in **Figure S12**, when voltage window of 0.01-2 V is applied, the CV curves experience much higher currents and an increasing anodic peak at around 0.5 V. While the curves are almost overlapped with small currents for the carbon fiber cycled between 0.1-2 V. The carbon fiber pieces are selected very carefully and their areas and weights are the same. Their capacities, as indicated in **Figure S13**, also vary greatly when the voltage windows are different. The carbon fiber cycled between 0.01-2 V shows capacity around four times as high as that of which cycled between 0.1-2 V. The influence to the cyclic stability of Bi-C is also nonnegligible when 0.01-2 V of voltage window is used. As can be seen in **Figure S14**,

despite the growing anodic peak with cycling at around 0.5 V that is from carbon fiber, the peaks associated with the reaction of sodium ion with bismuth also shift severely.

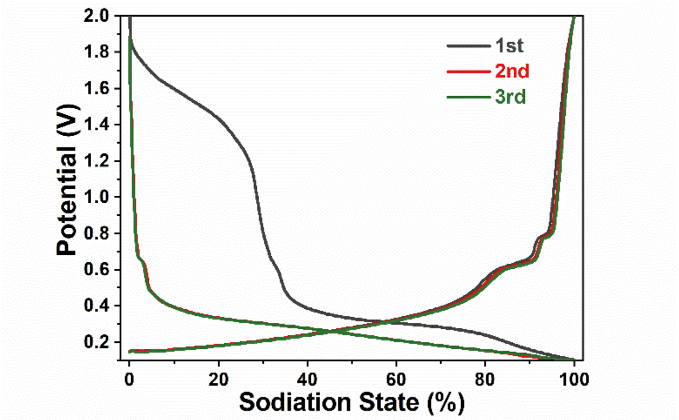

Figure S15. Initial three discharge-charge processes at 50 mA g^{-1} between 0.1-2 V of Bi-C/CF.

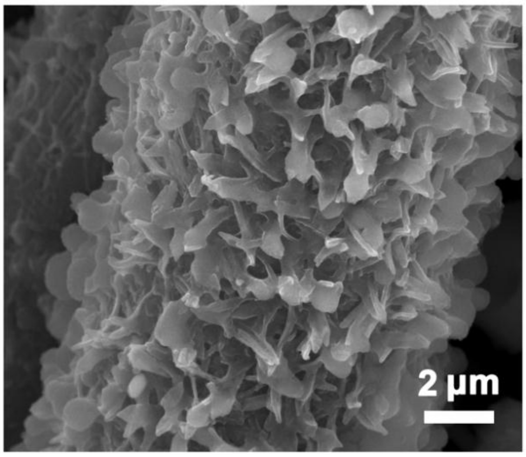

Figure S16. SEM images of Bi-C/CF electrode after 100 cycles at 50 mA g^{-1}.

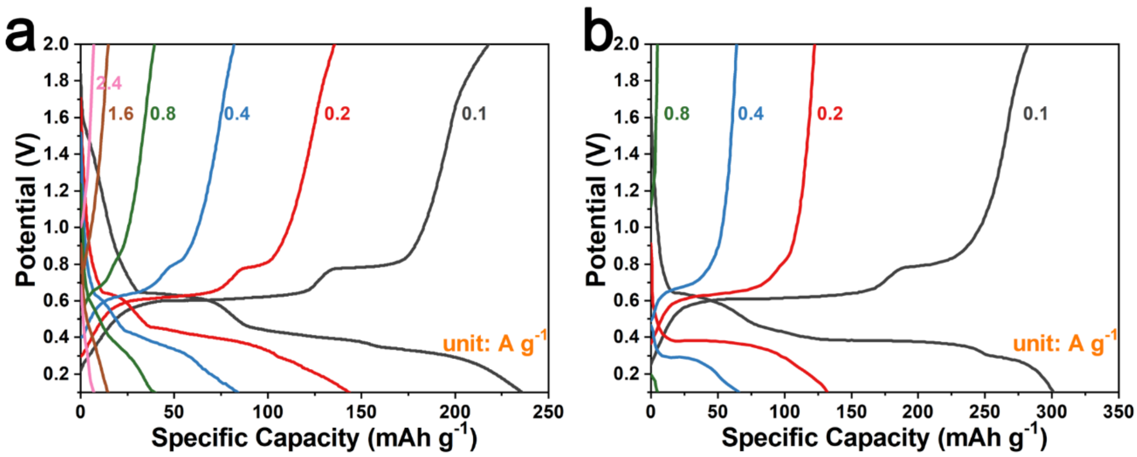

Figure S17. Discharge/charge profiles of Bi-C (a) and bare Bi (b) at different current densities.

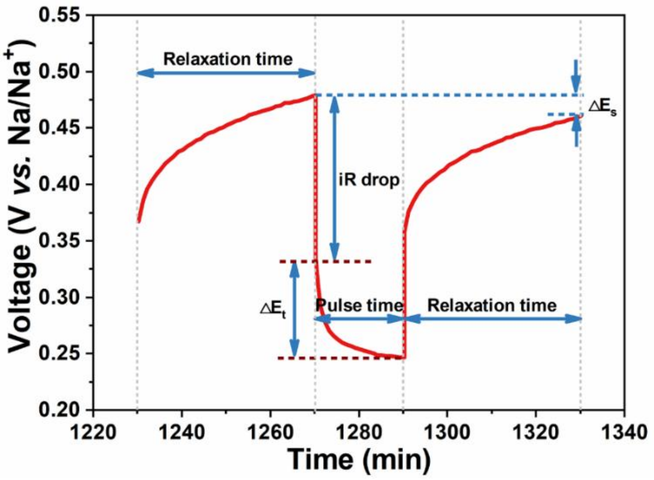

Figure S18. E *vs*. t profile for a single GITT during discharge process.

The sodium diffusion coefficients are calculated based on the following equation:[2]

$$D = \frac{4L^2}{\pi\tau}\left(\frac{\Delta E_s}{\Delta E_t}\right)^2$$

where *t* is the duration of the current pulse (s), τ is the relaxation time (s), ΔE_s is the steady-state potential change (V) by the current pulse, and ΔE_t is the potential change (V) during the constant current pulse after eliminating the *iR* drop. *L* is sodium ion diffusion length (cm); for compact electrode, it is equal to the thickness of electrode.

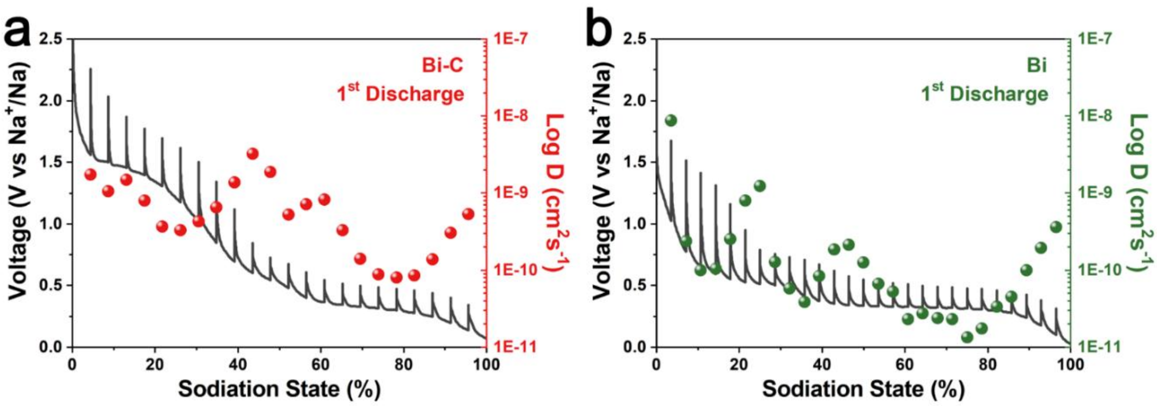

Figure S19. GITT curves and corresponding Na^+ diffusion coefficient at different sodiation states for Bi-C (a) and Bi (b).

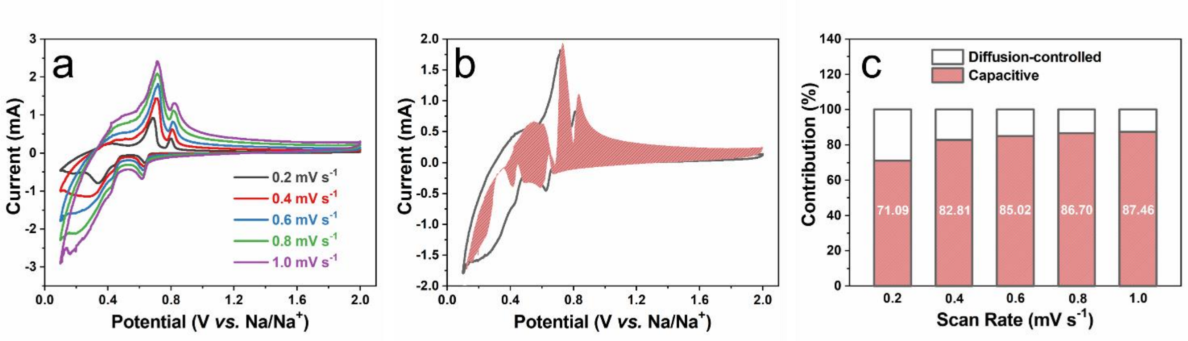

Figure S20. (a) CV curves at different scan rates from 0.2 to 1.0 mV s^{-1} for Bi-C/CF electrode. (b) Capacitive contribution (red pattern) at 0.6 mV s^{-1}. (c) Normalized capacitive and diffusion-controlled contribution ratios at different scan rates.

To evaluate the capacitive contribution to the overall capacity, CV curves at different scan rates are measured. As shown in Figure S20, the profiles display similar shapes at various scan rates. The contribution of diffusion-controlled or capacitive capacity can be verified by the following relationship between current (i) and scan rate (v):[3]

$$i = av^b$$

where a and b are adjustable parameters. b has the value ranging from 0.5 to 1. b = 0.5 implies the capacity is diffusion-controlled while b = 1 corresponds to capacitive behavior. The contribution of each part can be further quantified by the following equation:

$$i = k_1v + k_2v^{1/2}$$

where k_1 and k_2 are constants for a given potential. k_1v represents the capacitive contribution while $k_2v^{1/2}$ refers to the diffusion-controlled one. Figure S20b shows the calculated capacitive current (red pattern) in comparison with the measured current at scan rate of 0.6 mV s^{-1}. The capacitive contribution ratios at different scan rates are given in Figure S20c, which gradually enlarge as the scan rates increase. A high ratio of 87.46% can be obtained at 1 mV s^{-1}, implying the capacitive Na-storage occupies a great quantity of the whole capacity. The

abundant active sites at the boundaries between bismuth nanodots and nitrogen-doped carbon matrix can contribute to fast capacitive sodium storage.

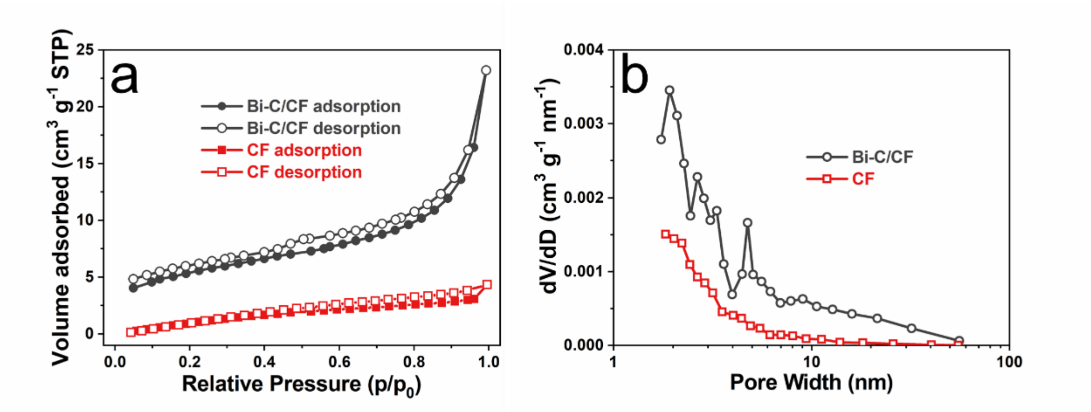

Figure S21. (a) Nitrogen adsorption-desorption isotherms of Bi-C/CF and blank carbon fiber. (b) Pore size distribution profiles of Bi-C/CF and blank carbon fiber.

Nitrogen adsorption-desorption measurements are performed for the Bi-C/CF and bare carbon fiber. The carbon fiber only possesses BET surface area of 7.1043 m^2 g^{-1}. For the Bi-C/CF sample, its adsorption-desorption curve displays type IV isotherm with type H3 hysteresis loop in the relative pressure range of 0.5-1.0 p/p_0.[4] The Bi-C/CF has overall surface area of 18.6645 m^2 g^{-1}. Considering the mass ratio of CF and Bi-C (> 10:1), the surface area is much enlarged by coating Bi-C arrays on the carbon fibers. In addition, the pore-size distribution calculated by Barrett-Joyner-Halenda (BJH) method demonstrate more pore volumes in the range of 2-10 nm for Bi-C/CF, which is beneficial for the electrolyte penetration and increases the contact area between active material and electrolyte.

References

[1] J. Qiu, S. Li, X. Su, Y. Wang, L. Xu, S. Yuan, H. Li, S. Zhang, *Chem. Eng. J.* **2017**, *320*, 300.

[2] D. T. Ngo, H. T. T. Le, C. Kim, J. Y. Lee, J. G. Fisher, I. D. Kim, C. J. Park, *Energy Environ. Sci.* **2015**, *8*, 3577.

[3] J. Wang, J. Polleux, J. Lim, B. Dunn, *J. Phys. Chem. C* **2007**, *111*, 14925.

[4] K. S. Sing, *Pure Appl. Chem.* **1985**, *57*, 603.
